# Fluorescent bead-based serological detection of *Toxoplasma gondii* infection in chickens

**DOI:** 10.1186/s13071-020-04244-6

**Published:** 2020-07-31

**Authors:** Benedikt T. Fabian, Fatima Hedar, Martin Koethe, Berit Bangoura, Pavlo Maksimov, Franz J. Conraths, Isabelle Villena, Dominique Aubert, Frank Seeber, Gereon Schares

**Affiliations:** 1 FG16: Mycotic and Parasitic Agents and Mycobacteria, Robert Koch-Institute, Berlin, Germany; 2 Friedrich-Loeffler-Institut, Federal Research Institute for Animal Health, Institute of Epidemiology, National Reference Centre for Toxoplasmosis, Greifswald-Insel Riems, Germany; 3 Faculty of Veterinary Medicine, Institute of Food Hygiene, Leipzig University, Leipzig, Germany; 4 Faculty of Veterinary Medicine, Institute of Parasitology, Leipzig University, Leipzig, Germany; 5 Department of Veterinary Sciences, Wyoming State Veterinary Laboratory, University of Wyoming, Laramie, USA; 6 EA 7510, UFR Medecine, University of Reims Champagne Ardenne, Reims, France; 7 Laboratory of Parasitology, National Reference Centre on Toxoplasmosis, Centre Hospitalier Universitaire de Reims, Reims, France

**Keywords:** *Toxoplasma gondii*, SAG1, Serum, Real-time PCR, Magnetic-Capture PCR, MAT, IFAT, ELISA, Luminex assay, Multiplexing

## Abstract

**Background:**

Free-ranging chickens are often infected with *Toxoplasma gondii* and seroconvert upon infection. This indicates environmental contamination with *T. gondii*.

**Methods:**

Here, we established a bead-based multiplex assay (BBMA) using the Luminex technology for the detection of *T. gondii* infections in chickens. Recombinant biotinylated *T. gondii* surface antigen 1 (TgSAG1_bio_) bound to streptavidin-conjugated magnetic Luminex beads served as antigen. Serum antibodies were detected by a fluorophore-coupled secondary antibody. Beads of differing color codes were conjugated with anti-chicken IgY or chicken serum albumin and served for each sample as an internal positive or negative control, respectively. The assay was validated with sera from experimentally and naturally infected chickens. The results were compared to those from reference methods, including other serological tests, PCRs and bioassay in mice.

**Results:**

In experimentally infected chickens, the vast majority (98.5%, *n* = 65/66) of birds tested seropositive in the BBMA. This included all chickens positive by magnetic-capture PCR (100%, *n* = 45/45). Most, but not all inoculated and TgSAG1_bio_-BBMA-positive chickens were also positive in two previously established TgSAG1-ELISAs (TgSAG1-ELISA_SL_, *n* = 61/65; or TgSAG1-ELISA_SH_, *n* = 60/65), or positive in an immunofluorescence assay (IFAT, *n* = 64/65) and in a modified agglutination test (MAT, *n* = 61/65). All non-inoculated control animals (*n* = 28/28, 100%) tested negative. In naturally exposed chickens, the TgSAG1_bio_-BBMA showed a high sensitivity (98.5%; 95% confidence interval, CI: 90.7–99.9%) and specificity (100%; 95% CI: 85.0–100%) relative to a reference standard established using ELISA, IFAT and MAT. Almost all naturally exposed chickens that were positive in bioassay or by PCR tested positive in the TgSAG1_bio_-BBMA (93.5%; 95% CI: 77.1–98.9%), while all bioassay- or PCR-negative chickens remained negative (100%; 95% CI: 85.0–100%).

**Conclusions:**

The TgSAG1_bio_-BBMA represents a suitable method for the detection of *T. gondii* infections in chickens with high sensitivity and specificity, which is comparable or even superior to other tests. Since assays based on this methodology allow for the simultaneous analysis of a single biological sample with respect to multiple analytes, the described assay may represent a component in future multiplex assays for broad serological monitoring of poultry and other farm animals for various pathogens.
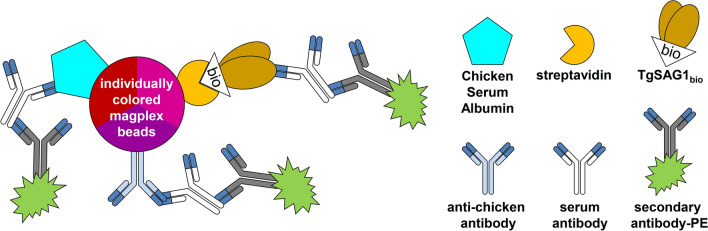

## Background

*Toxoplasma gondii* is a zoonotic protozoan parasite ranking among the most important foodborne pathogens worldwide [1–4]. Humans acquire toxoplasmosis either congenitally or postnatally [5]. *Toxoplasma gondii* can be transmitted congenitally from a recently infected mother to the fetus and may cause severe disease in children (e.g. hydrocephalus, seizures, mental or growth retardation) or even abortion. Congenitally infected children that are born without symptoms can also develop toxoplasmosis later in life (e.g. ocular toxoplasmosis). However, a large number of ocular uveitis cases in humans seem to be caused by postnatal *T. gondii* infections [6]. In most cases, postnatally acquired *T. gondii* infections, either through consumption of undercooked infected meat or by oral uptake of oocysts shed by felids, have no severe consequences [7]. Yet, persistent or recently acquired infections in immuno-compromised patients (e.g. transplant patients) may cause life-threatening disease [7].

Livestock animals are frequently infected by *T. gondii*, especially if they are reared free-ranging or have outdoor access [8]. In particular, free-range chickens are exposed to the environmental stage of the parasite, the oocysts, and the presence of cats on farm premises has been reported as a risk factor [9]. Although infections occur frequently, reports on clinically apparent toxoplasmosis in chickens are rare (reviewed by [10]). Due to the ground-feeding behavior of chickens and their susceptibility for *T. gondii*, they have been used as sentinels to monitor the potential contamination of farms with this parasite [11, 12]. The extent, to which chicken meat contributes to human infection with *T. gondii*, is unknown. There are specific dishes (e.g. chicken carpaccio, chicken sashimi and barbecued chicken) or products (sausages), in which the meat may not be sufficiently processed to inactivate the parasite. Moreover, handling raw chicken meat may represent a risk factor for human infection during cooking [13, 14]. Poor kitchen hygiene has also been reported as risk factor for human infection with the parasite [13].

In many epidemiological studies, serum or plasma were used to determine specific antibodies against *T. gondii.* The results have been used to estimate the burden of infection in chickens or on chicken farms (reviewed by [10]) to assess the potential risk for consumers [15], to identify chickens with viable *T. gondii* infections [16] or to assess risk factors for infection in this livestock species [9, 17–20]. Suitable serological techniques for chickens include MAT [12, 16, 21], IFAT [9, 21, 22] and ELISA [9, 21, 22].

In the present study, we aimed to establish a novel bead based multiplex assay (BBMA) applying the Luminex technology [23] for the detection of serum antibodies to *T. gondii* using recombinant biotinylated TgSAG1_bio_, a major tachyzoite surface antigen of this parasite [24]. We then validated the TgSAG1_bio_-BBMA against other well-established serological assays, i.e. the modified agglutination test (MAT), immunofluorescence assay (IFAT) and ELISAs, based on native TgSAG1, to detect *T. gondii* infection in chickens. To determine the diagnostic characteristics of the TgSAG1_bio_-BBMA, we used sera and tissues from experimentally or naturally infected chickens. These had been collected in previous studies [9, 22], in which we had determined the true infection status in these chickens using magnetic-capture-(MC-) real-time PCR (qPCR). Likewise, a combination of mouse-bioassay, MC-qPCR and quantitative PCR on acidic pepsin muscle digests (PD-qPCR) had been used.

Our results show that the TgSAG1_bio_-BBMA assay represents a suitable method with high sensitivity and specificity for the detection of *T. gondii* infections in chickens. Such bead-based assays provide an option for multiplexing because beads of numerous dye signatures (also called bead regions) are available. Thus, internal positive and background controls coupled to beads with different dye signatures can be evaluated simultaneously for each individual sample in the test. Moreover, the TgSAG1_bio_-BBMA allows combination with other serological markers, e.g. antigens from other pathogens, and has the potential to be included in future multiplex assays for large-scale sero-surveillance without a requirement for additional serum samples.

## Methods

### Parasite strains and experimental infections

We used samples from chickens (breed ISA JA 757) that had been experimentally infected with oocysts, tissue cysts or tachyzoites as reported in detail in a previous study [22]. Regardless of oocyst, tissue cyst or tachyzoite infections, the observation period usually lasted 5 weeks in all infected groups. In the case of tachyzoite infection, 6 inoculated and 6 non-inoculated birds were included and observed for a total of 10 weeks [22]. At the end of the observation period, blood was collected for serological analysis, the animals were euthanized and tissues (brain, heart, breast, thigh and drumstick musculature) were stored frozen at − 20 °C until further use. A total of 23 non-infected control chickens and 66 inoculated chickens were used, which were orally inoculated with oocysts or brains of chronically infected mice or by intravenous (i.v.) injection of *in vitro*-cultivated tachyzoites [22].

Three different *T. gondii* strains were used: the type II *T. gondii* strain CZ-Tiger [25]; type II *T. gondii* ME49 [26]; and type III *T. gondii* NED [27]. The CZ-Tiger strain parasites were already available as oocysts while ME49 and NED parasites were initially cultivated as tachyzoites [28] and passaged *via* CD-1 mice and cats to generate tissue cysts and oocysts, respectively [22].

For infecting chickens, three different doses of oocysts were applied, i.e. 1 × 10^3^ (CZ-Tiger, ME49 and NED), 1 × 10^5^ (CZ-Tiger and ME49), or 1 × 10^6^ oocysts per bird (CZ-Tiger and ME49) [22]. For tissue cyst infection, one microscopically-positive mouse brain per bird was inoculated orally [22]. *In vitro* cultivated tachyzoites (*T. gondii* NED, 1 × 10^6^ tachyzoites in 0.1 ml of sterile isotonic saline solution (B. Braun Melsungen AG, Melsungen, Germany)) were inoculated i.v. into the wing vein of each bird.

### Polymerase chain reaction

MC-qPCR was essentially performed as described [29] with some slight modifications [22]. For the PD-qPCR, tissues were digested [11, 30] and the qPCR performed on digests as described [31, 32] using primers and a probe targeting the 529 bp repeat of *T. gondii* [33].

### Sera and serological tests

#### Sera

Sera from experimentally and naturally exposed chickens were collected as detailed previously [9, 22]. When the chickens were sacrificed, blood was collected and allowed to clot. The samples were then centrifuged, sera collected and stored frozen at − 20 °C until further use.

#### MAT

The MAT for the detection of *T. gondii*-specific IgY antibodies was performed as previously described [34]. Each serum or fluid sample was two-fold serially diluted. A titer of 1:1 was applied as the positive cut-off.

#### IFAT

The IFAT was performed as reported previously [9]. Only complete peripheral fluorescence of the tachyzoite was considered specific. A titer of 1:50 was used as the positive cut-off.

#### TgSAG1-ELISA

Chicken sera were tested for antibodies against the native *T. gondii* tachyzoite surface antigen TgSAG1 as described [9] using affinity purified TgSAG1 of *T. gondii* tachyzoites [35, 36]. A cut-off optimized for maximum diagnostic specificity was applied (ELISA index 0.242) as previously described for the TgSAG1-ELISA_SH_ [9]. The subscript SH indicates “specificity high”. Moreover, a less-stringent cut-off optimized for Youden’s index was used (ELISA index 0.104) for the TgSAG1-ELISA_SL_ [9]. Here, the subscript “SL” indicates “specificity low”.

#### Luminex TgSAG1

Recombinant production of biotinylated TgSAG1 (TgSAG1_bio_) and coupling of the antigen to Luminex MagPlex® beads (Luminex Cooperation, ‘s-Hertogenbosch, The Netherlands) has been described recently [37]. In brief, the entire mature coding region of TgSAG1 (aa 31–289) was expressed as an N-terminal fusion with maltose binding protein (MBP), which enhances solubility during translation. MBP can be cleaved-off *in situ* by TEV protease, which recognizes its cleavage sequence and thus separates MBP from TgSAG1 in the engineered protein [37]. After that the putative GPI-attachment site (Gly289 of TgSAG1) at the C-terminus, a 4 kDa peptide sequence (AviTag) and a six histidine-tag were added and used for purification. The AviTag is recognized by *Escherichia coli* biotin ligase BirA, resulting in the C-terminal *in situ* biotinylation of TgSAG1 at a unique lysine residue within the tag sequence. Subsequently, biotinylated TgSAG1_bio_ was purified by metal chelate affinity chromatography using an Äkta Purifier system [37].

The chemical coupling to beads of either recombinant streptavidin (Sav; Anaspec, Fremont, CA, USA; 16.67 μg/10^6^ MagPlex® beads, region 34), chicken serum albumin (CSA, Sigma-Aldrich, Darmstadt, Germany; 12 µg/10^6^ MagPlex® beads, region 54) as a negative control, or chicken IgY (Jackson ImmunoResearch Laboratories, West Grove, PA, USA; 6.67 µg/10^6^ MagPlex® beads, region 52) as a positive control followed the instructions of the xMAP® Cookbook [38, 39]. Prior to coupling, bead stocks were vortexed for 30 s and sonicated for 30 s in a water-bath. Beads (1.5 × 10^6^) were transferred from the stock to individual reaction tubes for each of the three bead regions, i.e. dye signatures, washed with distilled water, vortexed and sonicated for a few seconds and incubated in 80 µl 0.1 M NaH_2_PO_4_, pH 6.2 per tube. The tubes were again vortexed and sonicated for 10 s prior to addition of 500 µg N-hydroxysulfosuccinimide (Sulfo-NHS; Thermo Fisher Scientific, Waltham, MA, USA) and 1-ethyl-3-[3-dimethylaminopropyl]carbodiimide hydrochloride (EDC; Thermo Fisher Scientific). The beads were then incubated for 20 min on a horizontal shaker (300× *rpm*) and vortexed briefly after 10 min.

After incubation, the tubes were again placed in a magnetic separator for 2 min and the supernatant removed. The beads were washed twice with 250 µl of 0.05 M 2-(N-morpholino) ethanesulfonic acid (MES; Sigma-Aldrich) before addition of conjugates, and each tube was adjusted to 500 µl by adding 0.05 M MES. Tubes were briefly vortexed and then incubated for 2 h on a horizontal shaker at 300× *rpm*, with an intermittent brief vortexing step after 1 h. The tubes were then placed in a magnetic separator for 2 min and the supernatant removed. Five hundred µl of PBS containing 0.02 % Tween-20, 0.1 % BSA and 0.05 % sodium azide (PBS-TBN) were added and the beads incubated for 30 min on a horizontal shaker at 300× *rpm*, before the samples were placed in a magnetic separator for 2 min to remove the supernatant. The beads were washed twice with 1 ml PBS-TBN without sonication. For storage, the beads were resuspended in 500 µl Stabilguard (Surmodics, Inc., Eden Prairie, MN, USA). TgSAG1_bio_ (10 ng/1500 beads) was added to the Sav-coated bead mix as described elsewhere [37].

Testing by BBMA was performed as previously described for human sera [39]. The 3 bead mixes were adjusted to 1000 beads per sample in PBS containing 1% BSA (PBS-B). Twenty µl of each region were added to 100 µl of samples (sera diluted 1:200 in PBS-B) in a 96-well plate (Greiner Bio-One, Kremsmünster, Austria). The plate, protected from light, was shaken at room temperature for 60 min. Beads were then washed twice with PBS containing 0.1 % Tween-20 (PBS-T). One hundred µl of rabbit-F(ab’)2 anti-chicken IgG-phycoerythrin (Rockland Immunochemicals, Limerick, PA, USA), diluted 1:333 in PBS-B, added to each sample and the plate shaken at room temperature for 30 min, protected from light. Beads were again washed twice, resuspended in 125 µl PBS-B and analysed with a Bio-Plex 200 reader (Bio-Rad, Hercules, CA, USA). The readout was set to 50 beads per region and the timeout was set to 90 s. The High RP1 target option was activated (i.e. increasing the voltage on the photomultiplier tube) for increased sensitivity, allowing quantification of lower concentrations of analytes and three wells containing only beads and PBS-B were set as blank samples.

### Mouse bioassay

The mouse bioassay was conducted as described [9]. Briefly, IFNɣ-knockout mice (GKO, IFNɣ -/-, C.129S7(B6)-Ifngtm1Ts/J) or IFNɣ-receptor-knockout mice (GRKO, IFNɣreceptor -/-; B6.129Sv/Ev-IfngrtmAgt) were used. The mice were inoculated with pepsin-digested [11, 30] heart and drumstick musculature (2 mice for each kind of tissue, monitored for 42 days).

### Statistical analysis

R version 3.5.3 (R Foundation for Statistical Computing, Vienna, Austria; http://www.R-project.org) and the R package *optimal.cutpoints* were used to define an optimal cut-off for the TgSAG1_bio_-BBMA and to determine diagnostic sensitivity, specificity, and positive and negative predictive values, including 95% confidence intervals (95% CI). In addition, diagnostic sensitivity and diagnostic specificity, including 95% CI, were determined using tools that were available online (http://vassarstats.net/clin1.html). To assess the overall diagnostic performance of the test, Youden’s index was calculated by the following formula using Excel spreadsheet functions: Sensitivity + Specificity – 1 [40]. To determine the relatedness of values measured in various serological diagnostic tests, linear regression was performed using the “lm” command in R, version 3.5.3. For this analysis, median fluorescence intensity (MFI) values and titers in IFAT and MAT were log_10_-transformed. Sera for which no titer had been determined in IFAT or MAT (i.e. seronegative sera), arbitrary titers of 1:25 (IFAT) or 1:0.5 (MAT) were used to allow for the calculation of log_10_ values.

Figures were assembled using R, version 3.5.3 or 4.0.0 (packages *ggplot2*, *reshape* and *scales*).

## Results

Based on the promising results obtained with the BBMA using human sera and the strong performance of TgSAG1_bio_ [37, 39], we strived for a transfer of this assay to animal species, including chickens, to establish an improved method for large scale, efficient serological monitoring. Although there is a number of BBMAs for veterinary purposes, these tests mainly focus on viral infections [41–44] and cannot be easily compared with our assay, which, to the best of our knowledge represents the first BBMA focusing on parasitic pathogens in chickens.

### Cut-off selection and diagnostic characteristics using sera from experimentally infected chickens

Sera collected from experimentally infected chickens were examined by the TgSAG1_bio_-BBMA (Additional file 1: Table S1). They had been collected from 23 non-infected and 66 infected chickens (orally inoculated with oocysts and brains of chronically infected mice, or intravenously with *in vitro*-cultivated tachyzoites, described in Methods). At the end of the observation periods, the infection state of the inoculated chickens was assessed in brain, heart, thigh, breast and drumstick musculature by MC-qPCR. Detailed results of these examinations were reported elsewhere [22].

For selecting an appropriate cut-off to score results as positive or negative by TgSAG1_bio_-BBMA, all inoculated chickens served as a positive reference population whereas non-infected control animals were defined as negative. Based on these assignments, an optimal Youden’s index was obtained when a median fluorescence intensity (MFI) of 322.5 was used as the cut-off. Relative to the reference standard, TgSAG1_bio_-BBMA showed a diagnostic sensitivity of 98.5% (95% CI: 91.8–100%; *n* = 65/66) and a diagnostic specificity of 100% (95% CI: 85.2–100%; *n* = 23/23) for the reference populations (Table 1).

**Table 1:** Summary of the characteristics of serological tests relative to two references of experimental chickens, (A) *T. gondii* inoculated *vs* non-inoculated chickens, (B) MC-qPCR (magnetic capture quantitative PCR) positive *vs* non-inoculated chickens, stratified for the tests. For the analysis, all experimentally inoculated chickens were excluded, if their infection had not been confirmed by MC-qPCR

Reference	Serological test	% diagnostic sensitivity [95% CI] (positive/reference positive^a^)	% diagnostic specificity [95% CI] (negative/reference negative^b^)	Youden’s index
(A) Inoculated *vs* non-inoculated				
	TgSAG1_bio_-BBMA	98.5 [90.7–99.9] (65/66)	100 [82.2–100] (23/23)	0.99
	TgSAG1-ELISA_SH_^c^	91.1 [80.6–96.3] (60/66)	100 [82.2–100] (23/23)	0.91
	TgSAG1-ELISA_SL_^c^	92.4 [82.5–97.2] (61/66)	100 [82.2–100] (23/23)	0.92
	IFAT^c^	97.0 [88.5–99.5] (64/66)	100 [82.2–100] (23/23)	0.97
	MAT^c^	92.4 [82.5–97.2] (61/66)	100 [82.2–100] (23/23)	0.87
(B) MC-qPCR positive *vs* non-inoculated				
	TgSAG1_bio_-BBMA	100 [90.4–100] (45/45)	100 [82.2–100] (23/23)	1.00
	TgSAG1-ELISA_SH_^c^	100 [90.4–100] (45/45)	100 [82.2–100] (23/23)	1.00
	TgSAG1-ELISA_SL_^c^	100 [90.4–100] (45/45)	100 [82.2–100] (23/23)	1.00
	IFAT^c^	100 [90.4–100] (45/45)	100 [82.2–100] (23/23)	1.00
	MAT^c^	100 [90.4–100] (45/45)	100 [82.2–100] (23/23)	1.00

^a^A chicken was regarded as reference-positive if at least one of the tissues from this animal tested positive by MC-qPCR

^b^All non-inoculated control chickens were regarded as reference-negative

^c^Results published previously [22] but shown for comparison

*Abbreviation*: CI, confidence interval

**Fig. 1:**
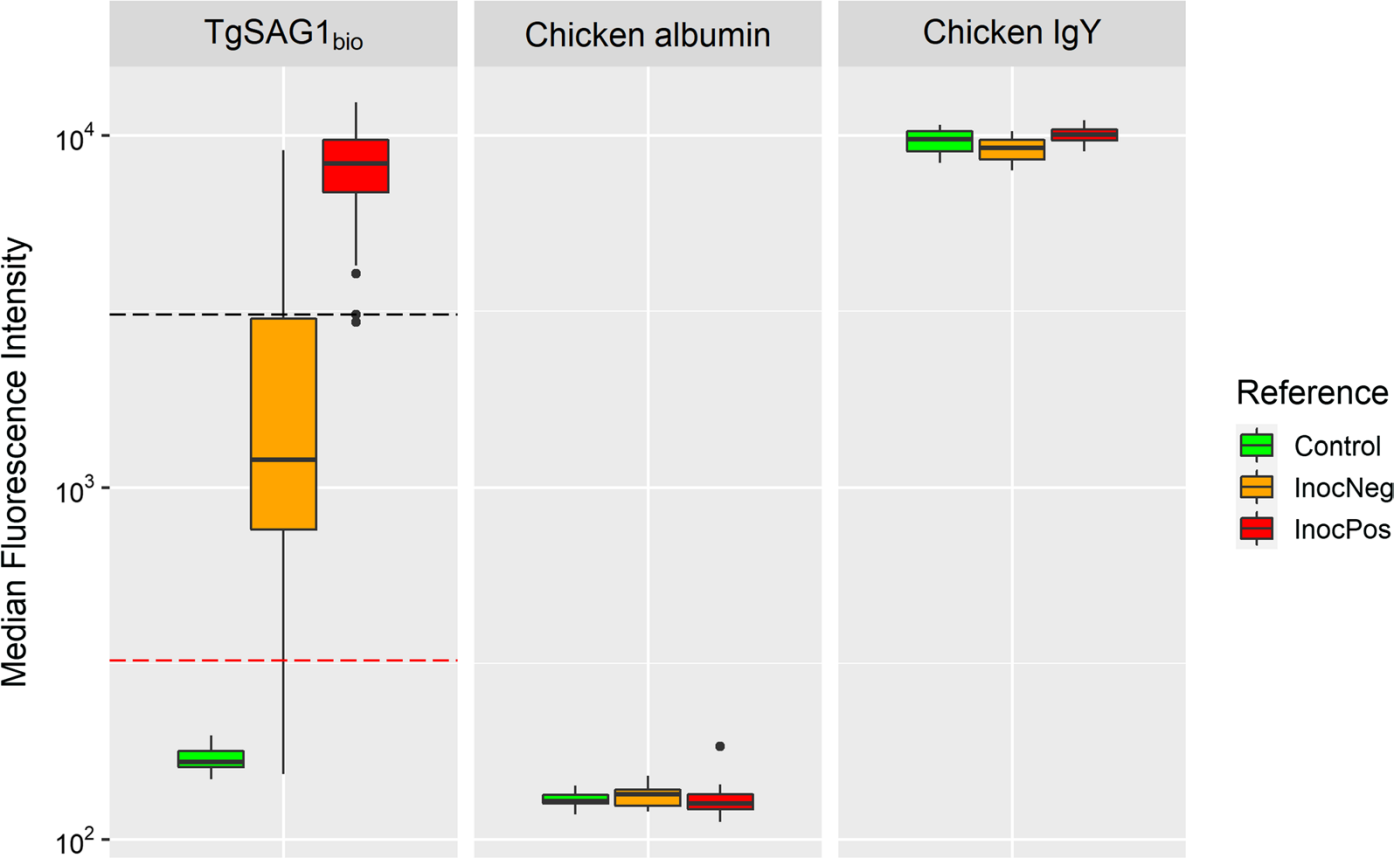
Performance of the TgSAG1_bio_ bead-based multiplex assay in experimentally *Toxoplasma gondii*-infected chickens. Median fluorescence intensities (MFI) in sera collected from non-inoculated chickens (green, *n* = 23) and inoculated chickens (*n* = 66). Results of inoculated chickens are split into inoculated and negative in direct detection by magnetic capture real time PCR (InocNeg, orange, *n* = 21) or inoculated and positive in direct detection (InocPos, red, *n* = 45). Data are displayed as box plots of median (line), 25th and 75th percentile (box), the 1.5-fold interquartile ranges (whiskers) and outliers (dots). The cut-off to separate reaction of inoculated from non-inoculated chickens is indicated by a red line (MFI = 322.5). The cut-off used within the group of inoculated chickens to separate the reactions of birds that tested positive by direct detection is indicated by a black line (MFI = 3092). In addition to the specific reactions (TgSAG1_bio_), results for internal controls, i.e. negative (chicken albumin; background) and positive controls (chicken IgY), are shown

Inoculated chickens that had tested positive by direct detection (MC-qPCR) showed higher MFI values than inoculated chickens, for which direct detection methods failed to confirm infection (Fig. 1). To separate MC-qPCR-positive chickens from MC-qPCR-negative chickens, a cut-off of MFI 3092 was optimal. At this cut-off, MC-qPCR-positive chickens were detected with a diagnostic sensitivity of 97.8% (95% CI: 88.2–99.9%; *n* = 44/45) and a diagnostic specificity of 76.2% (52.9–91.8%, *n* = 39/44). There were 5 serologically false-positive results (i.e. MC-qPCR negatives that tested serologically positive) and one false-negative finding (i.e. a MC-qPCR positive, testing serologically negative using MFI 3092 as the cut-off). The serum of the false-negative chicken showed a TgSAG1_bio_-BBMA MFI of 2939.

In experimentally infected chickens, the MFI values for CSA-loaded beads (negative control) were generally very low, while the MFI values were always high in chicken anti-IgY-loaded beads (positive or IgY concentration control) (Fig. 1).

### Comparison to other serological tests and MC-PCR in experimentally infected chickens

The results of the TgSAG1_bio_-BBMA were compared to those obtained by other antibody detection techniques (TgSAG1-ELISA_SH_; TgSAG1-ELISA_LH_, IFAT and MAT) reported previously [22]. Overall, the TgSAG1_bio_-BBMA detected the largest number of experimentally inoculated chickens (98.5%, 65/66) and was superior to the IFAT (97.0%, 64/66), followed by the TgSAG1-ELISA_SL_ and MAT (92.4%, 61/66) and the TgSAG1-ELISA_SH_ (90.9%, 60/66) (Table 1). All control animals were correctly identified as negative in all serological tests, including the TgSAG1_bio_-BBMA.

Among all inoculated chickens, only those inoculated with oocysts (89.5%; 34/38) or tissue cysts 68.8% (11/16) tested positive, when brain, heart, thigh, breast or drumstick tissue was examined by MC-qPCR. All these 45 MC-qPCR-positive birds also tested positive in the TgSAG1_bio_-BBMA using MFI 322.5 as a cut-off (Fig. 1). The other serological tests applied in this study showed a similar performance; all MC-PCR-positive chickens tested positive, while the control birds remained negative.

### Performance of TgSAG1_bio_-BBMA relative to other serological tests in naturally exposed chickens

To confirm the findings obtained with experimentally infected chickens, sera of naturally infected chickens were used (details on the selection of chickens have been reported elsewhere [9]). Sera were examined by TgSAG1_bio_-BBMA (Additional file 1: Table S2) and compared to results of other antibody detection techniques (ELISA, IFAT, MAT) or the results of direct parasite detection. Results of direct detection attempts were available for 59 of 61 chickens as previously reported [9].

Sera were also tested by the serological tests described above. Based on the results obtained with the majority of tests (i.e. excluding 18 of the initial 446 sera, for which half of the results were either positive or negative), an MFI of 483 was established as the optimal cut-off (optimal Youden’s index). Using this value, the TgSAG1_bio_-BBMA had a diagnostic sensitivity of 90.0% (95% CI: 78.2–96.7%; *n* = 45/50) and a diagnostic specificity of 98.9% (95% CI: 97.3–99.7%; *n* = 374/378) for the reference population. When a cut-off of MFI = 322.5, established for the experimentally infected chickens, was applied to the field chickens, the diagnostic characteristics were identical to those reported for the cut-off of MFI = 483; we thus decided to use MFI = 322.5 for further comparisons.

**Fig. 2:**
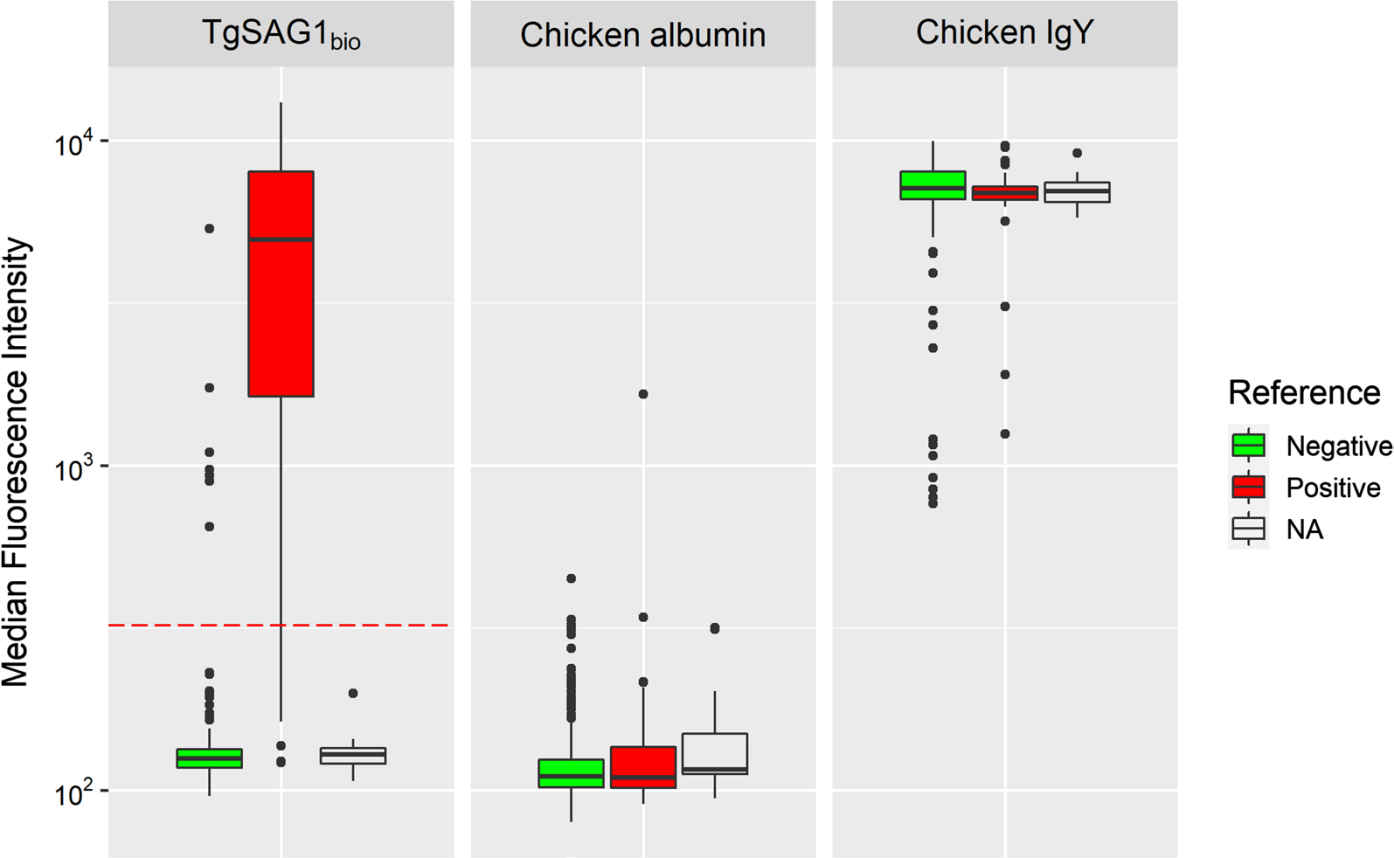
Performance of the TgSAG1_bio_-BBMA in chickens naturally exposed to *Toxoplasma gondii*. Median fluorescence intensity (MFI) in sera collected from reference-negative chickens (green, *n* = 378), reference-positive chickens (red, *n* = 50) and chickens that could not be assorted to the reference population (NA, *n* = 18) because of diverging results in serological reference assays (ELISA, IFAT and MAT). Data are displayed as box plots showing median (line), 25th and 75th percentile (box), the 1.5-fold interquartile ranges (whiskers) and outliers (dots). The cut-off applied is indicated by a red line (MFI = 322.5). In addition to the specific reactions (TgSAG1_bio_), also internal control reactions, i.e. negative control (chicken albumin) and a positive control reaction (chicken IgY) are shown. *Notes*: All negative reference chickens with background reactions MFI > 322.5 tested negative in the TgSAG1_bio_-BBMA. Two positive reference chickens with background reactions MFI > 322.5 were regarded as negligible, because their TgSAG1_bio_-BBMA reactions were at least 7-times higher and exceeded MFI 9000

A few sera (*n* = 5) showed background reactivity slightly above the cut-off (Fig. 2). However, this was only observed for two sera that scored TgSAG1_bio_-BBMA-positive (Fig. 2). Since the specific TgSAG1_bio_ reaction was 27- or 7-times higher in these sera than the background reaction, the latter was regarded negligible.

Some sera had up to 10-times less IgY than the majority of sera (Fig. 2; outliers). This was the case in 4 of the positive reference sera and 13 of the negative reference sera. As it was not clear, whether the reduced IgY content in these sera might have had an effect on the cut-off selection and the diagnostic characteristics, statistical analysis was repeated without these 17 samples. The analysis resulted in the same cut-off, a diagnostic sensitivity of 89.1% (95% CI: 76.4–96.4%; *n* = 41/46) and a diagnostic specificity of 98.9% (95% CI: 97.3–99.7%; *n* = 361/365).

### Diagnostic performance of TgSAG1_bio_-BBMA and other serological tests relative to direct detection in naturally exposed chickens

Relative to a reference standard of direct *T. gondii* detection (i.e. chickens with heart or drumstick tissues positive either by mouse bioassay, MC-qPCR or PD-qPCR), serological analysis by the TgSAG1_bio_-BBMA with the cut-off MFI = 322.5 showed the highest Youden’s index of all serological tests. This was also reflected by a high diagnostic sensitivity (93.5%, 29/31) and maximal diagnostic specificity (100%, 28/28) (Table 2). Only the MAT had the same diagnostic specificity (100%, 28/28), but at the same time a substantially lower diagnostic sensitivity (67.7%, 21/31) (Table 2).

**Table 2:** Characteristics of serological tests relative to mouse bioassay and PCR, MC-qPCR (magnetic capture quantitative PCR), PD-qPCR (conventional quantitative PCR on acid pepsin-digested tissues)

Serological test	% diagnostic sensitivity [95% CI] (positive/reference positive^a^)	% diagnostic specificity [95% CI] (negative/reference negative^a^)	Youden’s index
TgSAG1_bio_-BBMA	93.5 [77.1–98.9] (29/31)	100 [85.0–100] (28/28)	0.94
TgSAG1-ELISA_SH_^b^	83.9 [65.5–93.9] (26/31)	89.3 [70.6–97.2] (25/28)	0.73
TgSAG1-ELISA_SL_^b^	96.8 [81.5–99.8] (30/31)	60.7 [40.7–77.9] (17/28)	0.58
IFAT^b^	90.3 [73.1–97.5] (28/31)	82.1 [62.4–93.2] (23/28)	0.73
MAT^b^	67.7 [48.5–82.7] (21/31)	100 [85.0–100] (28/28)	0.68

^a^Chickens were regarded as reference-positive if at least one of the tissues (heart, drumstick) tested positive by one of the assays. The remaining chickens were regarded as reference-negative

^b^Results published previously [9], only shown for comparison

*Abbreviation*: CI, confidence interval

**Fig. 3:**
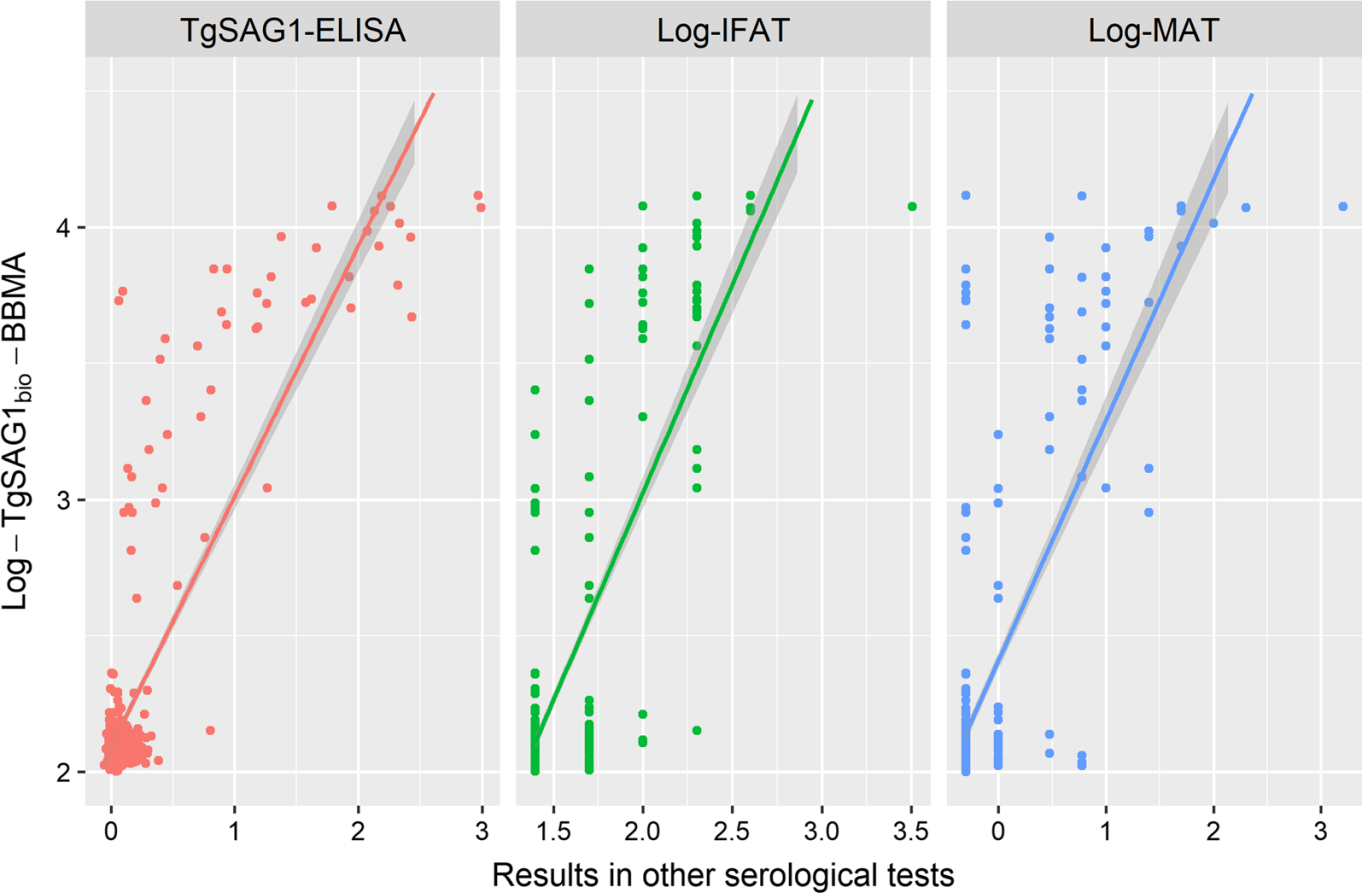
Linear regression analyses of fluorescence intensities measured in the TgSAG1_bio_-BBMA and the results of ELISA, IFAT and MAT. Each graph shows a linear regression line, including 95% confidence limits (grey). Log_10_-transformed MFI values in the TgSAG1_bio_-BBMA correlated highest to the ELISA indices in the TgSAG1-ELISA (Adjusted *R*^2^, 74.6%). In contrast, linear regression with log_10_-transformed IFAT titer (Log-IFAT) or log_10_-transformed MAT titers (Log-MAT) revealed adjusted *R*^2^ of 65.6% or 57.5%, respectively

The antigenic properties of recombinant TgSAG1_bio_ used in the BBMA may differ from those of native TgSAG1 used in ELISA or the complex antigens used in IFAT or MAT. The extent, to which the values obtained in the different tests were related to each other, was studied by linear regression. Log_10_-transformed MFI values in the TgSAG1_bio_-BBMA correlated better with the ELISA indices in the TgSAG1-ELISA (adjusted *R*^2^, 74.6%; *P* < 0.001) than with log_10_-transformed IFAT titers (adjusted *R*^2^, 65.6%; *P* < 0.001) or log_10_-transformed MAT titers (adjusted *R*^2^, 57.5%; *P* < 0.001) (Fig. 3).

## Discussion

In the present study, we used data and sera from previous studies to characterize the potential of a BBMA for assessing the serological response of chickens against *T. gondii*. To our knowledge, this is the first description of a *T. gondii*-specific serological BBMA for chickens. BBMAs have a number of advantages as compared to MAT, IFAT and ELISA. First, they allow for the simultaneous serological testing of antibodies directed against several pathogens by using a number of antigens coupled to beads with individual colour codes (also called regions). Moreover, with analytes coupled to different bead regions and added to the same BBMA one can implement internal standards, such as a control for sufficient IgY levels in a test sample. Such internal controls can often not be included in other serological assays such as ELISA, IFAT or MAT, when testing individual samples, or only at the expense of testing additional samples.

A similar approach for detecting *T. gondii* infections in animals other than chickens using BBMA has been reported [45, 46]. This assay used a *T. gondii* tachyzoite lysate as antigen. In contrast, we applied a bacterially expressed biotinylated recombinant TgSAG1 [37], the major surface antigen of *T. gondii* tachyzoites [24], which is used widely for serodiagnosis in humans, but also in wild or livestock animals (e.g. [47–55]). Our recombinant TgSAG1_bio_ is unique in that it allows an oriented and reproducible coupling of the antigen to streptavidin-coated magnetic Luminex beads *via* a single C-terminal biotin [37]. It thus adopts a similar orientation on the beads as native TgSAG1 does on the parasite surface through its GPI anchorage in the membrane [56], thereby exposing the major conformational epitope recognized by human antibodies to the solute [57]. However, it is not known, if this epitope is also important in chickens.

Only two other studies have reported the use of recombinant TgSAG1 for serodiagnosis in chickens [47, 58]. In both cases, TgSAG1 was used in a denatured form on immunoblots, leading to low sensitivity compared to other recombinant tachyzoite antigens or total lysate, respectively. In contrast, TgSAG1 used in our reference ELISA is purified from tachyzoite lysate by monoclonal antibody affinity chromatography [9]. We conclude that TgSAG1_bio_ as used here is as good as the native protein, but available in larger amounts with less effort.

Since the TgSAG1_bio_-BBMA was previously tested for human sera and showed excellent diagnostic characteristics in comparison to commercial diagnostic assays [37], we extended the use of the TgSAG1_bio_-BBMA to chicken sera. We compared the diagnostic characteristics not only with those of other serological tests, but also with the infection status of the birds as determined by samples from experimentally or naturally exposed chickens, by direct detection including conventional qPCR on pepsin-digested muscle tissues, MC-qPCR or mouse bioassay.

By using sera of experimentally inoculated chickens, a cut-off was established to separate *T. gondii*-inoculated from non-inoculated chickens. With this cut-off, the diagnostic performance of the TgSAG1_bio_-BBMA in experimental chickens was as good as or superior to that of the ELISAs, IFAT and MAT performed in comparison. Moreover, much higher MFI values were observed in *T. gondii* inoculated chickens that had tested positive by direct detection (MC-qPCR) than in inoculated chickens without directly detectable infection (Fig. 1). However, we cannot exclude that also inoculated animals without a positive MC-qPCR result were viably infected. Probably, *T. gondii* had multiplied better in MC-qPCR-positive animals, which may have resulted in a wider distribution of the parasite and could thus have increased the chance to detect the infection by MC-qPCR. At the same time, it may have led to increased exposure to parasitic antigens, including TgSAG1, and thus to a higher level of specific antibodies. In a previous study, it has been observed that higher antibody levels increased the likelihood of a positive result in MC-qPCR in sheep [29].

The cut-off established by using experimental chicken sera proved also suitable in naturally infected chickens. With this cut-off, it was possible to separate sera that had tested positive or negative in a number of reference tests including two ELISAs, IFAT and MAT with high diagnostic sensitivity and specificity. Moreover, especially the TgSAG1-ELISA indices, but also the titers in IFAT or MAT correlated significantly with the MFI values of the TgSAG1_bio_-BBMA.

In naturally exposed chickens that had also been examined by direct detection methods, the TgSAG1_bio_-BBMA showed both high diagnostic sensitivity and specificity to identify infected animals. The TgSAG1-ELISA_SL_ was the only test that had a higher diagnostic sensitivity compared to the TgSAG1_bio_-BBMA. However, its diagnostic specificity was much lower.

The second cut-off established in experimental chickens (MFI = 3092) allowed separating birds that were positive in direct detection from birds that were inoculated, but appeared negative in direct detection. In naturally exposed chickens, however, the assay failed to detect 29.0% (9/31) of the direct detection-positive chickens, including eight birds, in which the mouse bioassay had proven a viable *T. gondii* infection. Therefore, we do not recommend applying this second cut-off.

There is a number of reasons, why the findings obtained with experimentally infected chickens do not necessary match the results for naturally exposed birds: (i) infections in naturally exposed chickens may date back much further than the more recent infections in experimentally inoculated chickens. Therefore, the exposure to tachyzoite antigens and to TgSAG1 in particular may have occurred more recently in experimentally exposed chickens, which might have had the effect that antibody levels to this antigen are still higher. (ii) Experimentally inoculated chickens may have been exposed to higher parasite doses as compared to naturally exposed chickens. Even low oocyst numbers, which are still infective, but might result in a lower tachyzoite burden, may have induced the development of tissue cysts and eventually viable *T. gondii* infection in naturally exposed birds. The differences between experimentally and naturally exposed chickens show, that test development and validation in veterinary medicine should never rely on data obtained by experimental infections alone.

## Conclusions

The TgSAG1_bio_-BBMA correlated very well with other standard serological tests and was superior to these tests in detecting viable *T. gondii* infections in chickens. Since the TgSAG1_bio_-BBMA allows for multiplexing and the option for including internal controls as a prerequisite for standardization, it seems to be a promising test, which may also be adapted to further animal species [59]. Similar to previous work [45], the TgSAG1_bio_-BBMA may be adapted to pigs, also in combination with tests for other parasitic (e.g. *Trichinella*), bacterial (e.g. *Salmonella*) or viral pathogens (e.g. hepatitis E virus). As the recombinant antigen used in the TgSAG1_bio_-BBMA is readily available in large quantity and high purity, the test will be easy to standardize and the production of a large number of tests seems feasible. Moreover, the TgSAG1_bio_-BBMA has advantages over existing methods, some of which require large sample volumes, and is therefore particularly attractive in situations, where only minute sample volumes are available. At the same time, it is suitable for parallel testing against several pathogens for comprehensive serological monitoring.

## Supplementary information

**Additional file 1: Table S1.** Data table containing information and serological results on experimental chickens. **Table S2.** Data table containing information and serological results on naturally exposed chickens.

## Data Availability

Data supporting the conclusions of this article are included within the article and its additional files. The raw datasets used and analyzed during the present study are available from the corresponding author upon reasonable request.

## Abbreviations

BBMA: bead-based multiplex assay
BSA: bovine serum albumin
CSA: chicken serum albumin
ELISA: enzyme-linked immunosorbent assay
IFAT: immunofluorescent antibody test
MAT: modified agglutination test
MC-qPCR: magnetic-capture real time PCR
MFI: median fluorescence intensity
O.D.: optical density
NC: negative control
PBS: phosphate buffered saline
PBS-B: PBS containing 1% BSA
PBS-T: PBS containing 0.1% Tween-20
PBS-TBN: 0.02% Tween-20, 0.1% BSA and 0.05% sodium azide
PC: positive control
PD-qPCR: pepsin digest-based real time PCR
TgSAG1_bio_: surface antigen 1 of *Toxoplasma gondii* tachyzoites, biotinylated

## References

[1] Havelaar AH, van Rosse F, Bucura C, Toetenel MA, Haagsma JA, Kurowicka D (2010). Prioritizing emerging zoonoses in the Netherlands. PLoS ONE..

[2] Scallan E, Hoekstra RM, Angulo FJ, Tauxe RV, Widdowson MA, Roy SL (2011). Foodborne illness acquired in the United States - major pathogens. Emerg Infect Dis..

[3] Scallan E, Hoekstra RM, Mahon BE, Jones TF, Griffin PM (2015). An assessment of the human health impact of seven leading foodborne pathogens in the United States using disability adjusted life years. Epidemiol Infect..

[4] Torgerson PR, Mastroiacovo P (2013). The global burden of congenital toxoplasmosis: a systematic review. Bull World Health Organ..

[5] Schlüter D, Däubener W, Schares G, Gross U, Pleyer U, Lüder C (2014). Animals are key to human toxoplasmosis. Int J Med Microbiol..

[6] Maenz M, Schlüter D, Liesenfeld O, Schares G, Gross U, Pleyer U (2014). Ocular toxoplasmosis past, present and new aspects of an old disease. Prog Retin Eye Res..

[7] Robert-Gangneux F, Darde ML (2012). Epidemiology of and diagnostic strategies for toxoplasmosis. Clin Microbiol Rev..

[8] Stelzer S, Basso W, Silván JB, Ortega-Mora LM, Maksimov P, Gethmann J (2019). *Toxoplasma gondii* infection and toxoplasmosis in farm animals: risk factors and economic impact. Food Waterborne Parasitol..

[9] Schares G, Bangoura B, Randau F, Goroll T, Ludewig M, Maksimov P (2017). High seroprevalence of *Toxoplasma gondii* and probability of detecting tissue cysts in backyard laying hens compared with hens from large free-range farms. Int J Parasitol..

[10] Dubey JP (2010). Toxoplasmosis of animals and humans.

[11] More G, Maksimov P, Pardini L, Herrmann DC, Bacigalupe D, Maksimov A (2012). *Toxoplasma gondii* infection in sentinel and free-range chickens from Argentina. Vet Parasitol..

[12] Dubey JP, Lehmann T, Lautner F, Kwok OC, Gamble HR (2015). Toxoplasmosis in sentinel chickens (*Gallus domesticus*) in New England farms: seroconversion, distribution of tissue cysts in brain, heart, and skeletal muscle by bioassay in mice and cats. Vet Parasitol..

[13] Kapperud G, Jenum PA, Stray-Pedersen B, Melby KK, Eskild A, Eng J (1996). Risk factors for *Toxoplasma gondii* infection in pregnancy: results of a prospective case-control study in Norway. Am J Epidemiol..

[14] Cook AJC, Gilbert RE, Buffolano W, Zufferey J, Petersen E, Jenum PA (2000). Sources of Toxoplasma infection in pregnant women: European multicentre case-control study. Br Med J..

[15] Dubey JP, Hill DE, Jones JL, Hightower AW, Kirkland E, Roberts JM (2005). Prevalence of viable *Toxoplasma gondii* in beef, chicken, and pork from retail meat stores in the United States: risk assessment to consumers. J Parasitol..

[16] Dubey JP, Laurin E, Kwowk OC (2016). Validation of the modified agglutination test for the detection of *Toxoplasma gondii* in free-range chickens by using cat and mouse bioassay. Parasitology..

[17] Zhu J, Yin J, Xiao Y, Jiang N, Ankarlev J, Lindh J (2008). A sero-epidemiological survey of *Toxoplasma gondii* infection in free-range and caged chickens in northeast China. Vet Parasitol..

[18] Millar PR, Alves FMX, Teixeira VQ, Vicente RT, Menezes EM, Sobreiro LG (2012). Occurrence of infection with*Toxoplasma gondii* and factors associated with transmission in broiler chickens and laying hens in different raising systems. Pesqui Vet Bras..

[19] Magalhaes FJ, da Silva JG, Ribeiro-Andrade M, Pinheiro JWJ, Aparecido Mota R (2016). High prevalence of toxoplasmosis in free-range chicken of the Fernando de Noronha Archipelago, Brazil. Acta Trop..

[20] Salant H, Yasur-Landau D, Baneth G, Spira DT, Hamburger J (2016). A seroprevalence study of *Toxoplasma gondii* in some bird and animal species of Israel and its possible reflection on environmental contamination. Isr J Vet Med..

[21] Casartelli-Alves L, Boechat VC, Macedo-Couto R, Ferreira LC, Nicolau JL, Neves LB (2014). Sensitivity and specificity of serological tests, histopathology and immunohistochemistry for detection of *Toxoplasma gondii* infection in domestic chickens. Vet Parasitol..

[22] Schares G, Koethe M, Bangoura B, Geuthner AC, Randau F, Ludewig M (2018). *Toxoplasma gondii* infections in chickens - performance of various antibody detection techniques in serum and meat juice relative to bioassay and DNA detection methods. Int J Parasitol..

[23] Graham H, Chandler DJ, Dunbar SA (2019). The genesis and evolution of bead-based multiplexing. Methods..

[24] Burg JL, Perelman D, Kasper LH, Ware PL, Boothroyd JC (1988). Molecular analysis of the gene encoding the major surface antigen of *Toxoplasma gondii*. J Immunol..

[25] Jurankova J, Opsteegh M, Neumayerova H, Kovarcik K, Frencova A, Balaz V (2013). Quantification of *Toxoplasma gondii* in tissue samples of experimentally infected goats by magnetic capture and real-time PCR. Vet Parasitol..

[26] Lunde MN, Jacobs L (1983). Antigenic differences between endozoites and cystozoites of *Toxoplasma gondii*. J Parasitol..

[27] Howe DK, Sibley LD (1995). *Toxoplasma gondii* comprises three clonal lineages: correlation of parasite genotype with human disease. J Infect Dis..

[28] Geuthner AC, Koethe M, Ludewig M, Pott S, Schares G, Daugschies A (2014). Persistence of *Toxoplasma gondii* tissue stages in poultry over a conventional fattening cycle. Parasitology..

[29] Opsteegh M, Langelaar M, Sprong H, den Hartog L, De Craeye S, Bokken G (2010). Direct detection and genotyping of *Toxoplasma gondii* in meat samples using magnetic capture and PCR. Int J Food Microbiol..

[30] Dubey JP (1998). Refinement of pepsin digestion method for isolation of *Toxoplasma gondii* from infected tissues. Vet Parasitol..

[31] Legnani S, Pantchev N, Forlani A, Zini E, Schares G, Balzer J (2016). Emergence of cutaneous neosporosis in a dog receiving immunosuppressive therapy: molecular identification and management. Vet Dermatol..

[32] Schares G, Herrmann DC, Maksimov P, Matzkeit B, Conraths FJ, Moré G (2017). Chicken line-dependent mortality after experimental infection with three type IIxIII recombinant *Toxoplasma gondii* clones. Exp Parasitol..

[33] Talabani H, Asseraf M, Yera H, Delair E, Ancelle T, Thulliez P (2009). Contributions of immunoblotting, real-time PCR, and the Goldmann-Witmer coefficient to diagnosis of atypical toxoplasmic retinochoroiditis. J Clin Microbiol..

[34] Dubey JP, Desmonts G (1987). Serological responses of equids fed *Toxoplasma gondii* oocysts. Equine Vet J..

[35] Hosseininejad M, Azizi HR, Hosseini F, Schares G (2009). Development of an indirect ELISA test using a purified tachyzoite surface antigen SAG1 for sero-diagnosis of canine *Toxoplasma gondii* infection. Vet Parasitol..

[36] Maksimov P, Buschtöns S, Herrmann DC, Conraths FJ, Görlich K, Tenter AM (2011). Serological survey and risk factors for *Toxoplasma gondii* in domestic ducks and geese in Lower Saxony. Germany. Vet Parasitol..

[37] Klein S, Stern D, Seeber F. Expression of *in vivo* biotinylated recombinant antigens SAG1 and SAG2A from *Toxoplasma gondii* for improved seroepidemiological bead-based multiplex assays. Under review. 2020 (preprint at https://www.researchsquare.com/article/rs-33963/v1; 10.21203/rs.3.rs-33963/v1). 10.1186/s12896-020-00646-7 PMC7542104 33023547

[38] Angeloni S, Cordes R, Dunbar S, Garcia C, Gibson G, Martin C (2016). xMAP® Cookbook.

[39] Garg M, Stern D, Gross U, Seeberger PH, Seeber F, Varon Silva D (2019). Detection of anti-*Toxoplasma gondii* antibodies in human sera using synthetic glycosylphosphatidylinositol glycans on a bead-based multiplex assay. Anal Chem..

[40] Youden D (1950). Index for rating diagnostic tests. Cancer..

[41] Watson DS, Reddy SM, Brahmakshatriya V, Lupiani B (2009). A multiplexed immunoassay for detection of antibodies against avian influenza virus. J Immunol Methods..

[42] Wang H, Cong F, Guan J, Xiao L, Zhu Y, Lian Y (2018). Development of a sensitive and specific xMAP assay for detection of antibodies against infectious laryngotracheitis and bronchitis viruses. Virol J..

[43] Wang H, Cong F, Guan J, Xiao L, Zhu Y, Lian Y (2019). Establishment of xMAP for the simultaneous detection of antibodies to Newcastle disease virus and avian influenza virus. Poult Sci..

[44] Germeraad E, Achterberg R, Venema S, Post J, de Leeuw O, Koch G (2019). The development of a multiplex serological assay for avian influenza based on Luminex technology. Methods..

[45] Bokken GC, Bergwerff AA, van Knapen F (2012). A novel bead-based assay to detect specific antibody responses against *Toxoplasma gondii* and *Trichinella spiralis* simultaneously in sera of experimentally infected swine. BMC Vet Res..

[46] Bokken GC, Portengen L, Cornelissen JB, Bergwerff AA, van Knapen F (2015). Bayesian estimation of diagnostic accuracy of a new bead-based antibody detection test to reveal *Toxoplasma gondii* infections in pig populations. Vet Parasitol..

[47] Appiah-Kwarteng C, Saito T, Toda N, Kitoh K, Nishikawa Y, Adenyo C (2019). Native SAG1 in *Toxoplasma gondii* lysates is superior to recombinant SAG1 for serodiagnosis of *T. gondii* infections in chickens. Parasitol Int..

[48] Velmurugan GV, Tewari AK, Rao JR, Baidya S, Kumar MU, Mishra AK (2008). High-level expression of SAG1 and GRA7 gene of *Toxoplasma gondii* (Izatnagar isolate) and their application in serodiagnosis of goat toxoplasmosis. Vet Parasitol..

[49] Kimbita EN, Xuan X, Huang X, Miyazawa T, Fukumoto S, Mishima M (2001). Serodiagnosis of *Toxoplasma gondii* infection in cats by enzyme-linked immunosorbent assay using recombinant SAG1. Vet Parasitol..

[50] Chong CK, Jeong W, Kim HY, An DJ, Jeoung HY, Ryu JE (2011). Development and clinical evaluation of a rapid serodiagnostic test for toxoplasmosis of cats using recombinant SAG1 antigen. Korean J Parasitol..

[51] Sudan V, Tewari AK, Singh H (2015). Serodiagnosis of *Toxoplasma gondii* infection in bovines from Kerala, India using a recombinant surface antigen 1 ELISA. Biologicals..

[52] Ferreira SCM, Torelli F, Klein S, Fyumagwa R, Karesh WB, Hofer H (2019). Evidence of high exposure to *Toxoplasma gondii* in free-ranging and captive African carnivores. Int J Parasitol Parasites Wildl..

[53] Seltmann A, Schares G, Aschenborn OHK, Heinrich SK, Thalwitzer S, Wachter B (2020). Species-specific differences in *Toxoplasma gondii*, *Neospora caninum* and *Besnoitia besnoiti* seroprevalence in Namibian wildlife. Parasit Vectors..

[54] Holec-Gasior L (2013). *Toxoplasma gondii* recombinant antigens as tools for serodiagnosis of human toxoplasmosis: current status of studies. Clin Vaccine Immunol..

[55] Tzanidakis N, Maksimov P, Conraths FJ, Kiossis E, Brozos C, Sotiraki S (2012). *Toxoplasma gondii* in sheep and goats: seroprevalence and potential risk factors under dairy husbandry practices. Vet Parasitol..

[56] Seeber F, Dubremetz JF, Boothroyd JC (1998). Analysis of *Toxoplasma gondii* stably transfected with a transmembrane variant of its major surface protein, SAG1. J Cell Sci..

[57] Graille M, Stura EA, Bossus M, Muller BH, Letourneur O, Battail-Poirot N (2005). Crystal structure of the complex between the monomeric form of *Toxoplasma gondii* surface antigen 1 (SAG1) and a monoclonal antibody that mimics the human immune response. J Mol Biol..

[58] Hotop A, Buschtöns S, Bangoura B, Zöller B, Koethe M, Spekker-Bosker K (2014). Humoral immune responses in chickens and turkeys after infection with *Toxoplasma gondii* by using recombinant antigens. Parasitol Res..

[59] Christopher-Hennings J, Araujo KP, Souza CJ, Fang Y, Lawson S, Nelson EA (2013). Opportunities for bead-based multiplex assays in veterinary diagnostic laboratories. J Vet Diagn Invest..

